# Delivery of IL-35 by *Lactococcus lactis* Ameliorates Collagen-Induced Arthritis in Mice

**DOI:** 10.3389/fimmu.2018.02691

**Published:** 2018-11-20

**Authors:** Massimo Maddaloni, Irina Kochetkova, Carol Hoffman, David W. Pascual

**Affiliations:** ^1^Department of Infectious Diseases and Immunology, University of Florida, Gainesville, FL, United States; ^2^Department of Microbiology and Immunology, Montana State University, Bozeman, MT, United States

**Keywords:** *Lactococcus*, probiotic, IL-35, therapeutic, IL-10, cytokines, regulatory T cells, CCR6

## Abstract

IL-35, a relatively newly discovered cytokine belonging to the larger IL-12 family, shows unique anti-inflammatory properties, believed to be associated with dedicated receptors and signaling pathways. IL-35 plays a pivotal role in the development and the function of both regulatory B (Bregs) and T cells (Tregs). In order to further its therapeutic potential, a dairy *Lactococcus lactis* strain was engineered to express murine IL-35 (LL-IL35), and this recombinant strain was applied to suppress collagen-induced arthritis (CIA). Oral administration of LL-IL35 effectively reduced the incidence and disease severity of CIA. When administered therapeutically, LL-IL35 abruptly halted CIA progression with no increase in disease severity by reducing neutrophil influx into the joints. LL-IL35 treatment reduced IFN-γ and IL-17 3.7- and 8.5-fold, respectively, and increased IL-10 production compared to diseased mice. Foxp3^+^ and Foxp3^−^ CD39^+^ CD4^+^ T cells were previously shown to be the Tregs responsible for conferring protection against CIA. Inquiry into their induction revealed that both CCR6^+^ and CCR6^−^ Foxp3^+or−^ CD39^+^ CD4^+^ T cells act as the source of the IL-10 induced by LL-IL35. Thus, this study demonstrates the feasibility and benefits of engineered probiotics for treating autoimmune diseases.

## Introduction

Rheumatoid arthritis (RA) is a chronic, inflammatory, systemic autoimmune disease that affects about 0.24% of the worldwide population, and roughly 1% of the general population in Western countries. RA is two to three times more common in women than in men (1–5). RA manifests as a chronic synovitis and progressive destruction of the joints, leukocyte infiltrates, and cartilage destruction and bone erosion. Approximately half of the afflicted patients become disabled over the progression of this disease (6). RA is mediated predominantly by CD4^+^ T cells overexpressing proinflammatory cytokines, particularly in the joints (7).

In order to test the efficacy of potential RA therapeutics and understand mechanisms of disease, the collagen-induced arthritis (CIA) model is often used (8). CIA is typically induced by immunizing rodents with bovine or chick type II collagen together with an adjuvant. This combination leads to immune attack of the host's native collagen involving components of both the innate and adaptive immune systems. Emphasis on regulating proinflammatory cytokines, particularly TNF-α, is key to minimizing disease since TNF-α can be detected in joints of RA patients (9, 10). Mouse CIA shares several clinical, histopathological and immunological features with human RA. Clinical features include erythema, edema, synovitis, pannus formation, and CD4^+^ T cell-mediated inflammation with extensive cartilage and bone damage, resulting in joint deformities (11–13). These similarities are commonly exploited to use CIA as a model for RA and as a tool to investigate novel approaches to prevent and treat RA. Current treatments focus on neutralizing TNF-α action via anti-TNF-α mAbs and TNF-α receptor antagonists (14, 15); however, such interventions have been problematic, making patients more susceptible to opportunistic infections (14–16). Hence, alternatives that can restore tolerance need to be sought.

In view of reducing autoimmunity, the use of probiotics can restore immune homeostasis to reduce autoimmunity (17–19). Historically, lactic acid bacteria (LAB) represented the core of probiotic-based interventions, although more recently nonpathogenic *E. coli* (20–22), attenuated *Salmonella* (23, 24), *Bifidobacterium spp*. (25), and some yeasts like *Saccharomyces boulardii* (26) also proved to be valuable tools as novel therapeutic and prophylactic interventions. Traditional molecular genetics, coupled with synthetic biology, provides an ample selection of promoters and terminators resulting in dynamic expression levels. Protein synthesis can be induced *in vitro* under nisin controlled expression (NICE), or use a promoter that is silent during *in vitro* culture, and only active *in vivo* subsequent infection of the host (27–29). LABs are considered ideal vectors for oral or mucosal delivery since they are inherently nonpathogenic, and they can survive the harsh conditions of the gastric environment. LABs are amenable to recombinant expression of passenger antigens (Ags) to stimulate immunity against a number of pathogens (30–32), to curb the effects of inflammatory bowel disease (33, 34), to control the proliferation of cancer cells (35), and to use for enzyme replacement therapy (36) among other applications (27, 37, 38). Currently, the only microbiota-based therapy that is FDA-approved and commercially available is fecal microbiota transplant (FMT) to treat *Clostridium difficile* infections. However, close to 200 microbiome-based therapeutics and diagnostics are currently in development (39).

The delivery of oral therapeutics represents a significant advantage of adapting LABs. In this context, we developed recombinant *Lactococcus lactis* (LL) for oral delivery to treat autoimmune disease (40). In a similar fashion, the studies described here focus on the expression of the immunosuppressive cytokine, IL-35. Oral administration of probiotic-based therapeutics is considered ideal because the gastrointestinal (GI) tract is home to T cells that can be stimulated to become Tregs and to seed other mucosal and systemic immune compartments. Another advantage of using genetically-modified (GM) probiotics is that these have been shown to be both effective and safe (37, 41–43). Our previous work has shown that an engineered LL derived from an industrial dairy strain can ferment commercial milk to a yogurt-like product, and when applied for treatment of CIA, can maintain the same therapeutic properties as when grown on a synthetic medium (40). IL-35 belongs to the IL-12 cytokine family [rev. in (44)]. This heterodimeric cytokine is composed of IL-12p35 and IL-27EBI3 and, in contrast to most members of the IL-12 family, has potent anti-inflammatory attributes. This property is mediated via IL-35 binding both IL-12Rβ2 chain and gp130, which results in specific triggering of STAT1 and STAT4 on T cells (45) and IL-12Rβ2 and IL-27Rα on B cells (46). IL-35 is immunosuppressive for a number of autoimmune disease models including CIA (47, 48), experimental autoimmune encephalomyelitis (49, 50), uveitis (46), type 1 diabetes (51), inflammatory bowel disease (IBD, and psoriasis (52).

CCR6 was previously shown to be expressed by Tregs (53), particularly those expressing RoRγt (54, 55). These Tregs have been shown involved in suppressing autoimmune diseases (56–58). CCR6^+^ Tregs have been found more commonly associated with human Tregs (55, 59), but CCR6 has also been found to be induced in mice subjected to CIA (55).

Given its potency to treat various autoimmune diseases (46–52), we queried the effectiveness of live vector delivery of IL-35. To accomplish this objective, murine IL-35 was expressed in *L. lactis* subsp lactis IL1403 (LL-IL35), and tested for its ability to ameliorate CIA. Results show that LL-IL35 is highly effective in treating CIA via the stimulation of CCR6^+^ and CCR6^−^ Tregs producing IL-10 and suppressing the proinflammatory cytokines, IL-17 and IFN-γ.

## Materials and methods

### Bacterial strain engineering and maintenance

*Lactococcus lactis* subsp. lactis IL1403 (IL1403) was grown on M17 plus 0.5% glucose (M17G). Microbiology work was performed according to NIH guidelines. Initial attempts to express IL-35 under the control of the constitutive p23 promoter yielded only rearranged, nonfunctional clones which confirmed the notion that IL-35 is difficult to express and to stabilize in a wide panel of hosts [our unpublished observations; (44, 48)]. To express IL-35, a synthetic gene codon-optimized for LL was designed in-house and then synthesized by Genscript (Piscathawa, NJ). The fragment contains an optimal Shine-Dalgarno sequence properly spaced from the ATG start codon, the usp45 secretion signal, the p35 coding region, a short flexible linker, the EBI3 coding region, and AgeI and SmaI sites at both ends. The fragment was excised with AgeI, gel-purified and cloned into pMSP3535H3 (53; a kind gift of Dr. DA Mills, University of California, Davis) yielding a construct named pBzMM150 (LL-IL35). Expression was achieved under the control of the nisin-inducible promoter borne on the vector.

### Collagen-induced arthritis (CIA)

All the animal experiments described in the present study were conducted in strict accordance with the recommendations in the Guide for the Care and Use of Laboratory Animals of the National Institutes of Health. All animal studies were conducted under protocols approved by Montana State University's and the University of Florida's Institutional Animal Care and Use Committee.

C57BL/6 males (B6; 8- to 10-weeks of age; Charles River Laboratories, Horsham, PA USA) were maintained at Montana State University Animal Resources Center or the University of Florida Animal Center Services. Groups of B6 males were induced with CIA using 100 μg of chicken collagen II (CII; Chondrex, Redmond, WA USA) emulsified in complete Freund's adjuvant (CFA) and administered s.c. as previously described (48, 60, 61). To treat CIA, mice were first orally gavaged with sterile 50% saturated sodium bicarbonate solution to neutralize stomach acidity, followed by 5 × 10^8^ CFUs of LL vector or LL-IL35, or vehicle only, sterile PBS. Two dosing regimens were tested, three doses administered on days 14, 21, and 28, and two doses given on days 18 and 25 post-CII challenge. Clinical scores were measured in a double-blind fashion after treatment, and mice were monitored to day 40. Each of the four limbs was evaluated using a scale of 0–3 (48, 60, 61): 0, no clinical signs; (1) mild redness of a paw or swelling of single digits; (2) significant swelling of ankle or wrist with erythema; (3) severe swelling and erythema of multiple joints; maximum score per mouse is 12.

### Cytokine elisa

CD4^+^ T cells were cell-sorted by negative selection on magnetic beads (Invitrogen, Grand Island, NY USA) from axillary, popliteal, and inguinal lymph nodes (LNs) yielding purity >98%. Purified CD4^+^ T cells (3 × 10^6^/ml) were restimulated with 5 μg/ml plate-bound anti-CD3 mAb (eBioscience, San Diego, CA USA) plus 5 μg/ml of soluble anti-CD28 mAb (eBioscience) for 48–72 h at 37°C and 5% CO_2_ similar to that previously described (48). Culture supernatants were collected for cytokine-specific ELISAs (48, 60, 61).

### Flow cytometry

Splenic and LN cells were stained with fluorochrome-labeled mAbs to CD4, CD39, Ly-6G, Ly-6C, CD11b, and Foxp3 (eBioscience, San Diego, CA USA), TGF-ß (R&D Systems, Minneapolis, MN USA), and fluorochrome-conjugated streptavidin (BD Pharmingen, San Jose, CA USA). For flow cytometry of Tregs, whole splenic and LN cells (5 × 10^6^/ml culture) were restimulated overnight with 50 μg/ml of CII (T-Cell Proliferation; Chondrex). The next day, cells were stimulated with 25 ng/ml PMA and 1 μg/ml ionomycin for an additional 3 h. Cells were harvested, washed, stained and analyzed as previously described (48, 60, 61).

To measure inflammatory cells in the arthritic joints, isolated limb joints were digested with 2 mg/ml collagenase (*Clostridium histolyticum*,Type IV; Sigma-Aldrich, St. Louis, MO) for 30 min at 37°C, and cell suspensions passed through a 70 μm cell strainer similar to that previously described (40, 62). Leukocytes were stained and analyzed by forward and side-scatter plots for Ly-6G^+^ Ly-6C^+^ CD11b^+^ neutrophils.

### Statistics

Mann-Whitney *U*-test was applied to statistically analyze clinical scores. The difference in arthritis incidence between experimental groups was checked with Fisher's exact probability test. One-way ANOVA was performed to analyze ELISA and flow cytometry results. Data were considered statistically significant, if *p*-value was < 0.05.

## Results and discussion

RA is a chronic, systemic autoimmune disorder affecting millions of patients in the US. Treatment of this progressive, degenerative disease demands constant use of anti-inflammatory drugs and often immunosuppressive treatments that increase susceptibility to infections and neoplasia (4, 15, 16). Instead, intervention strategies that focus on redirecting or reeducating T cell responses to produce tolerance instead of inflammation have the potential of being a superior treatment for RA.

**Figure 1:**
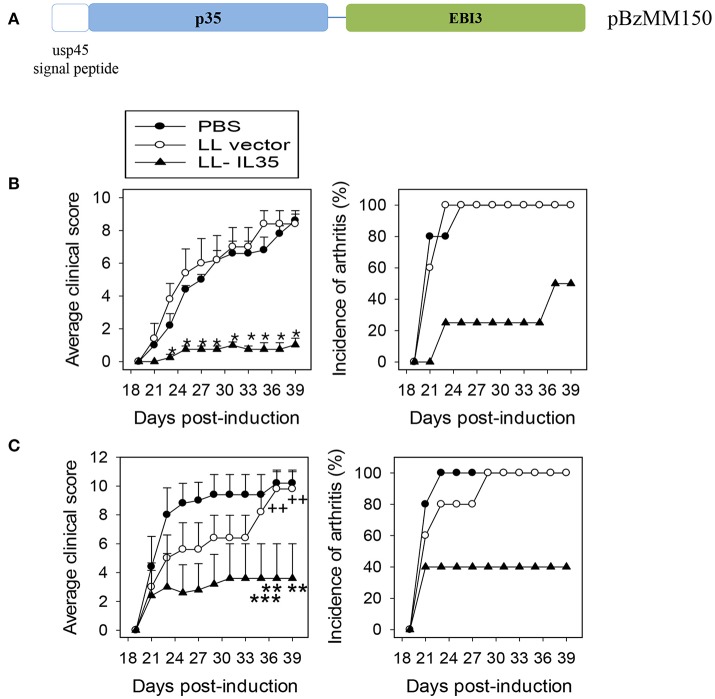
IL-35 inhibits CIA progression following treatment with LL-IL35. **(A)** Schematic map of the synthetic DNA used to construct pBzMM150 for murine IL-35 expression of IL-35 in *L. lactis*. The synthetic insert encodes in order: the usp45 secretion peptide genetically fused in-frame to the p35 subunit, a short flexible linker, fused to the EBI3. The synthetic DNA also features an optimal Shine-Dalgarno (SD) sequence at the optimal distance from the ATG initiation codon, in addition to the SD sequence present in the vector. The nisin-inducible promoter and a transcription terminator are borne on the expression vector pMSP3535H3. **(B,C)** CIA was induced in groups of C57BL/6 males with chick CII emulsified in complete Freund's adjuvant. Two regimens were tested: orally treated with 5 × 10^8^ CFUs of LL vector or LL-IL35 or sterile PBS on **(B)** 14, 21, and 28 days or **(C)** 18 and 25 days post-induction. Average clinical score per treatment group (left panels) represents severity of the disease, and incidence of arthritis depicts percent mice with affected joints in each treatment group (right panels). The sum of 10 mice/group is shown: **(B)** ^*^p < 0.001 vs. PBS-dosed or LL vector-treated mice, and **(C)** ^**^p < 0.02, ^***^p < 0.05 vs. PBS-dosed mice and ^++^*p* < 0.02 vs. LL vector-treated mice.

To address the void for such tolerance induction, we queried whether a probiotic LAB engineered to express the potent anti-inflammatory cytokine, IL-35 (Figure 1A), would diminish arthritis. Expression of IL-35 by LL-IL35 was detected by Western blot analysis using a rabbit polyclonal serum against an MBP-IL-35 fusion protein [produced in-house; (61)]. To test the therapeutic properties of LL-IL35, mice were challenged on day 0 with CII to induce CIA. Given its similarity, CIA is often exploited as an investigative tool to test novel strategies and therapeutics to prevent and treat RA. These mice were randomly divided into three groups for oral treatment: LL-IL35 (pBzMM150), LL vector (pMSP3535H3), or sterile PBS. Two treatment paradigms were tested: beginning intervention on day 14 resembling *Salmonella*-CFA/I treatment (60) with two additional doses on days 21 and 28 (Figure 1B) or beginning intervention at disease onset on day 18, followed by a second dose on day 25 (Figure 1C). Clinical scores were performed in double blind, and followed until day 39 post-induction. We generally do not see changes in disease severity beyond 39 days post-CII challenge.

Using the three-dose regimen, 50% of the LL-IL35-treated mice showed no symptoms and the remaining 50% developed minor symptoms as opposed to PBS- or LL vector-treated mice, who all developed severe arthritis by day 24 post-CII challenge. Notably, the severity of disease symptoms was significantly less (*p* < 0.001) in the LL-IL35-treated mice exhibiting an average clinical score of 1 in contrast to PBS- or LL vector-treated mice eventually achieving clinical scores of ~9 (Figure 1B). To test if LL-IL35 is effective in arresting the disease after disease onset, additional groups of CIA mice were treated using a two-dose regimen on days 18 and 25. Under this treatment, 40% of the LL-IL35-treated mice developed CIA vs. 100% of those treated with PBS or LL vector (Figure 1C). Compared to the three-dose regimen, the disease severity was greater for the LL-IL35-treated CIA mice subjected to the two-dose regimen, although significantly less (*p* < 0.05) when compared to similarly treated PBS- or LL vector-dosed mice. These data show that LL-IL35 can effectively reduce the symptoms of arthritis and the incidence of disease via its immunosuppressive capacity. Moreover, these results show that fewer doses of IL-35 delivered by LL are needed to curtail arthritis when compared to treatment with soluble protein (47, 48, 51, 52).

**Figure 2:**
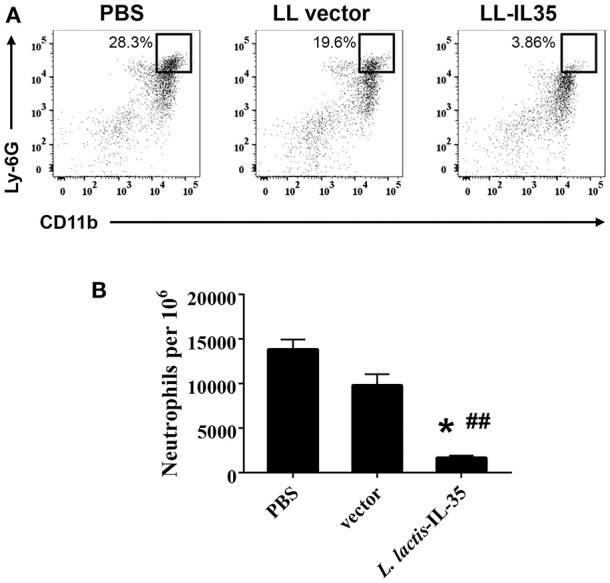
Oral LL-IL35 confers protection against CIA via reduction of neutrophil influx into joints. Reduced clinical scores and disease incidence described in Figure 1B are attributed to reduced neutrophil infiltration into the joints of LL-IL35-treated mice when compared to PBS-dosed and LL vector-treated mice. **(A)** Cell suspensions were analyzed by flow cytometry for Ly-6G^+^ CD11b^+^ neutrophils, and **(B)** quantified per 10^6^ cells. The depicted plots are representative of 5 mice/group; ^*^p < 0.001 vs. PBS-dosed mice; ^##^*p* < 0.005 vs. LL vector-treated mice.

Analysis of knee joints was performed to determine the extent of neutrophil infiltration. In agreement with these clinical findings, the LL-IL35-treated mice had markedly reduced Ly-6G^+^ CD11b^+^ cells (neutrophils) infiltrating the joints (Figure 2A) representing a 7- and 5-fold reduction compared to PBS-dosed or LL vector-treated groups, respectively (Figure 2B). Hence, IL-35 can reduce inflammation of the joints in CIA-challenged mice.

**Figure 3:**
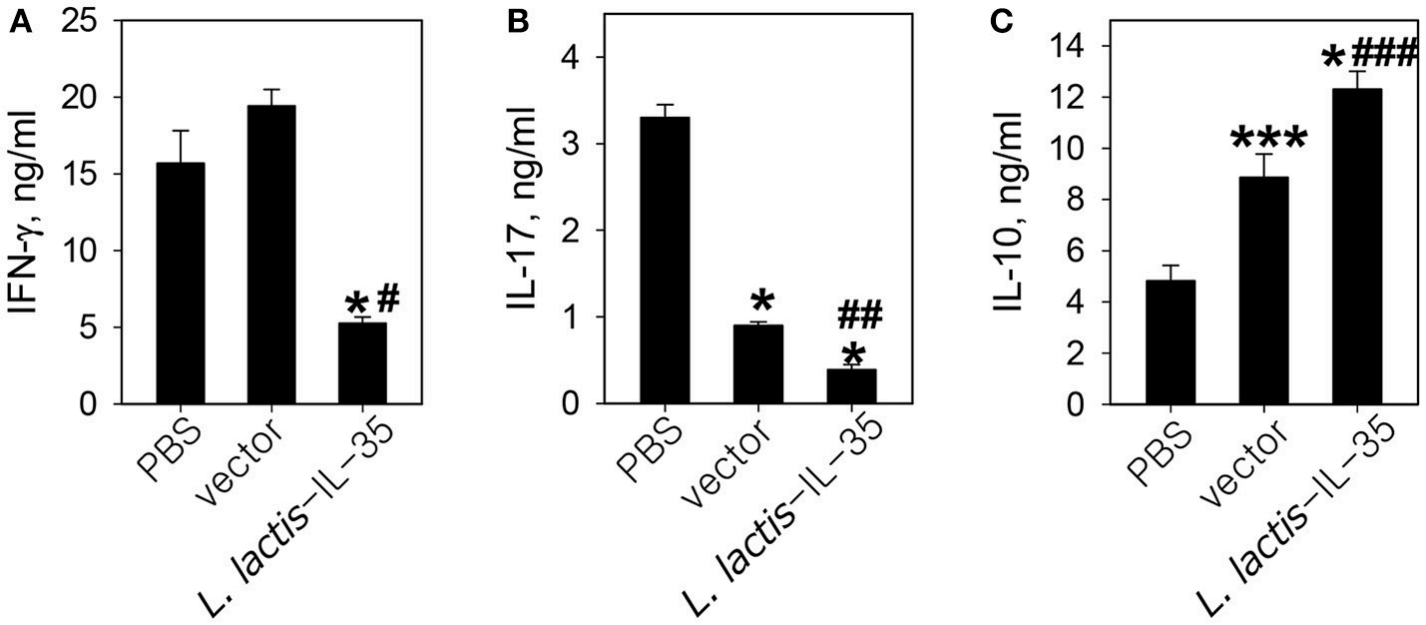
LL-IL35 reduces proinflammatory cytokines, IFN-γ and IL-17, with a concomitant increase in anti-inflammatory IL-10 production. CD4^+^ T cells were purified from PBS- or LL-IL35-dosed mice that received three treatments as described in Figure 1B. The LN CD4^+^ T cells were stimulated with plate-bound anti-CD3 and soluble anti-CD28 for 48–72 h, and collected supernatants were analyzed for **(A)** IFN-γ, **(B)** IL-17, and **(C)** IL-10 production. Depicted are the means ± SD of triplicate cultures as assessed by cytokine-specific ELISA; ^*^p < 0.001, ^***^p < 0.05 vs. PBS-dosed mice; ^#^*p* < 0.001, ^##^*p* < 0.01, ^###^*p* < 0.05 vs. LL vector-treated mice.

To investigate the possible mechanism of protection conferred by LL-IL35, CD4^+^ T cells purified from draining LNs were anti-CD3 + anti-CD28-restimulated and analyzed for cytokine production. These CD4^+^ T cells were obtained from mice dosed three times with PBS, LL vector, or LL-IL35 as described in Figure 1B. IFN-γ levels remained elevated between PBS-dosed and LL vector-treated mice, in contrast to LL-IL35-treated mice showing 3- to 3.7-fold reduction (*p* < 0.001; Figure 3A). Concomitantly, IL-17 levels were significantly less (*p* < 0.001) by 3.7- and 8.5-fold for LL vector and LL-IL35-treated groups, respectively, relative to PBS-dosed mice (Figure 3B). Moreover, treatment with LL-IL35 significantly reduced IL-17 by 2.3-fold compared to LL vector-treated mice (*p* < 0.01). Minimal stimulation of IL-10 was detected in the restimulated CD4^+^ T cells from the PBS-dosed mice (Figure 3C). In contrast, CD4^+^ T cells from LL vector- and LL-IL35-treated groups showed significantly increased IL-10 production (*p* < 0.05) by 1.8- and 2.5-fold, respectively. The difference between the LL vector- and LL-IL35-treated groups was significant (*p* < 0.05; Figure 3C) as well. The stimulation of IL-10 by LL vectors has been reported by others (56, 57). However, IL-10 induced by the LL vector-treated group was insufficient to suppress disease progression (Figure 1) and IL-17 production (Figure 3B). IL-10's importance for suppressing CIA was previously demonstrated in IL-10^−/−^ mice with CIA being refractive to IL-35 treatment (48), supporting the notion here of IL-10's relevance to CIA mice treated with LL-IL35. IL-35 has also been shown to stimulate IL-10 production (40, 47–49).

We have found that CD39^+^ CD4^+^ T cells are the primary Tregs responsible for resolving CIA (48, 60, 61). CD39 is an ectonucleoside triphosphate diphosphohydrolase-1which hydrolyzes ATP into AMP, thus quenching inflammatory signaling by extracellular ATP (58, 63). We also showed that CD25^+^ Tregs remained a subset of CD39^+^ CD4^+^ T cells, and that CD39 encompassed all of the Treg subsets (60). In fact, CD39^+^ Tregs were protective against CIA (40, 60). These Tregs are composed of two subsets, Foxp3^+^ and Foxp3^−^, and are interchangeable (60). Analysis of induction of CD39^+^ Tregs by the LL vector revealed no increase in the percentage of these Tregs in CIA mice (40), and CIA had only a modest impact upon their induction (40, 61).

**Figure 4:**
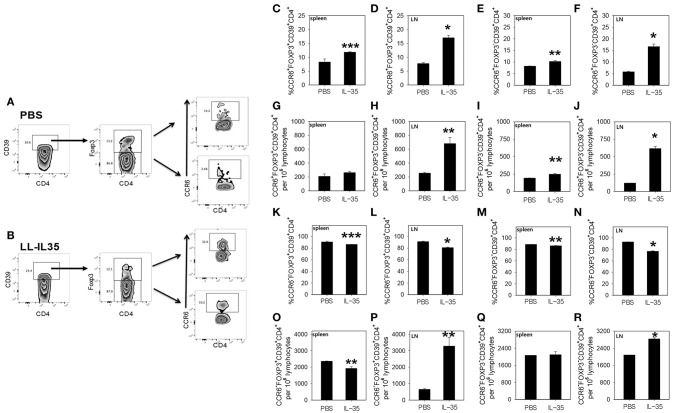
LL-IL35 induces CCR6^+^ and CCR6^−^ CD39^+^ CD4^+^ T cells in CIA mice. At the termination of the study, whole splenic and LN lymphocytes were restimulated with 50 μg/ml CII overnight, and then subjected to a short-term of PMA + ionomycin. LN CD39^+^ CD4^+^ T cells from **(A)** PBS-dosed and **(B)** LL-IL35-treated mice were gated on Foxp3^+^ and Foxp3^−^ cells, and analyzed for **(C–R)** for CCR6 expression by **(C,E,G,I,K,M,O,Q)** splenic and **(D,F,H,J,L,N,P,R)** LN lymphocytes. **(C,D)** Frequency of CCR6^+^ Foxp3^+^ and **(E,F)** CCR6^+^ Foxp3^−^ and absolute **(G,H)** CCR6^+^ Foxp3^+^ and **(I,J)** CCR6^+^ Foxp3^−^ T cells are shown. **(K,L)** Frequency of CCR6^−^ Foxp3^+^ and **(M,N)** CCR6^−^ Foxp3^−^ and absolute **(O,P)** CCR6^−^ Foxp3^+^ and **(Q,R)** CCR6^−^ Foxp3^−^ T cells are also shown. Depicted are the means ± SEM of 5 mice/group; ^*^p < 0.001, ^**^p ≤ 0.010, and ^***^p < 0.05 compared with PBS-dosed mice.

To examine the types of Tregs induced by LL-IL35 treatment, whole splenic and draining LN lymphocytes were cultured overnight with CII, and then pulsed with PMA + ionomycin to ascertain the type of Tregs induced in PBS-dosed and LL-IL35-treated mice. Lymphocytes were then stained for CD39, Foxp3, and CCR6 to identify the Treg subsets. Since CCR6 has been shown to be expressed by Tregs (53–55), we queried whether such Tregs may be induced as a consequence of IL-35 treatment. CD39^+^ CD4^+^ T cells were evaluated for expression of Foxp3 and CCR6 (Figures 4A,B). Upon examination of splenic Tregs derived from PBS-dosed mice compared to those present in LL-IL35-treated mice, a modest increase (*p* < 0.05) in the frequency, but not the total number of CCR6^+^ Foxp3^+^ CD39^+^ CD4^+^ T cells, was observed (Figures 4C,G). A modest difference (*p* ≤ 0.01) was also observed in the frequency and total number of splenic CCR6^+^ Foxp3^−^ CD39^+^ CD4^+^ T cells when compared to the PBS-dosed mice (Figures 4E,I). However, when similar analysis was performed for Tregs obtained from the draining LNs, a 2.2-fold increase in the frequency (*p* < 0.001) of CCR6^+^ Foxp3^+^ CD39^+^ CD4^+^ T cells was stimulated by LL-IL35 treatment compared to those present in PBS-dosed CIA mice (Figure 4D). The total number of these LN Tregs was also significantly (*p* ≤ 0.01) increased by 2.7-fold (Figure 4H). Subsequent analysis was performed on LN CCR6^+^ Foxp3^−^ CD39^+^ CD4^+^ T cells, and both the frequency and total number increased significantly by 2.9- (*p* ≤ 0.01) and 5.1-fold (*p* < 0.001), respectively (Figures 4F,J). These studies demonstrate that indeed CCR6^+^ Tregs are induced by IL-35 treatment of CIA mice.

Additional analyses were performed on both CCR6^−^ Foxp3^+^ and CCR6^−^ Foxp3^−^ CD39^+^ CD4^+^ T cells (Figures 4K–R). Examination of the splenic CCR6^−^ Foxp3^+^ CD39^+^ CD4^+^ T cells revealed that both the frequency and total number were modestly and significantly (*p* < 0.05) reduced for the LL-IL35-treated mice (Figures 4K,O). The frequency of splenic CCR6^−^ Foxp3^−^ CD39^+^ CD4^+^ T cells was slightly and significantly (*p* ≤ 0.01) reduced (Figure 4M), but the total number of these CD39^+^ CD4^+^ T cells showed no difference between PBS-dosed and LL-IL35-treated CIA mice (Figure 4Q). Similar analysis was also performed for the LN CCR6^−^ Foxp3^+^ and CCR6^−^ Foxp3^−^ CD39^+^ CD4^+^ T cells from the same treated CIA mice. While a slight reduction in the frequency of LN CCR6^−^ Foxp3^+^ CD39^+^ CD4^+^ T cells was observed for LL-IL35-treated mice (Figure 4L), the total number of CCR6^−^ Tregs was significantly (*p* ≤ 0.01) elevated by 5-fold (Figure 4P). Examination of the frequency of LN CCR6^−^ Foxp3^−^ CD39^+^ CD4^+^ T cells also showed a modest, but significant (*p* < 0.001) reduction in LL-IL35-treated mice relative to PBS-dosed mice (Figure 4N), but the total number of these LN T cells was significantly (*p* < 0.001) enhanced by 36% (Figure 4R). Hence, these analyses demonstrate that IL-35 treatment stimulates diverse subsets of Tregs including both CCR6^+^ and CCR6^−^ Tregs. Future studies will need to consider the longevity of these subsets for protection against CIA.

**Figure 5:**
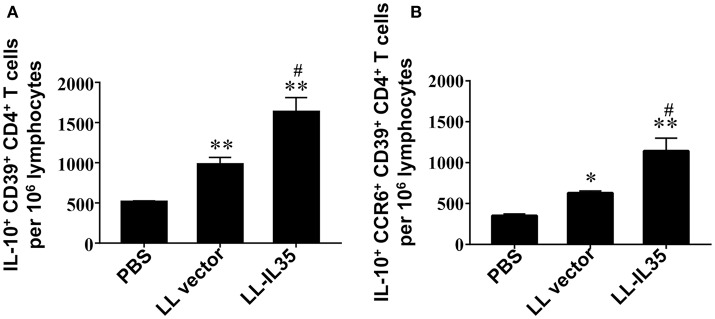
LL-IL35 stimulates IL-10 production by CD39^+^ Tregs and CCR6^+^ CD39^+^ Tregs. CIA mice treated with PBS, LL vector, or LL-IL35 as described in Figure 4. Intracellular IL-10 was measured for **(A)** CD39^+^ CD4^+^ and **(B)** CCR6^+^ CD39^+^ CD4^+^ T cells. Depicted are the means ± SEM of 5 mice/group; ^*^p < 0.001, ^**^p < 0.01 vs. PBS-dosed mice; ^#^*p* < 0.01 vs. LL vector-treated mice.

Inquiring into the activity of these LN CD39^+^ Tregs, analysis for IL-10 production was performed (Figure 5). Intracellular IL-10 measurements were conducted first for all CD39^+^ CD4^+^ T cells (both Foxp3^+^ and Foxp3^−^). The LL-IL35-treated mice showed 3.2- and 1.7-fold more IL-10-producing cells (*p* ≤ 0.01) than PBS-dosed and LL vector-treated CIA mice, respectively (Figure 5A). LL vector-treated mice showed 1.9-fold increase in the number of IL-10-producing CD39^+^ CD4^+^ T cells compared to PBS-dosed mice (*p* ≤ 0.01; Figure 5A). Examination of IL-10^+^ CCR6^+^ CD39^+^ CD4^+^ T cells (both Foxp3^+^ and Foxp3^−^) revealed that two-thirds of the total IL-10-producing cells induced by LL-IL35 treatment of CIA mice were derived from the CCR6^+^ subset (Figure 5B). The CCR6^+^ CD39^+^ CD4^+^ T cells induced with LL-IL35 resulted in significant 3.2- and 1.8-fold increase in IL-10-producing cells than those present in PBS-dosed (*p* ≤ 0.01) and LL vector-treated CIA mice (*p* < 0.01), respectively. LL vector-treated mice showed 1.8-fold increase in the number of IL-10-producing CCR6^+^ CD39^+^ CD4^+^ T cells compared to PBS-dosed mice (*p* ≤ 0.01; Figure 5B). These findings suggest that indeed both Foxp3^+^ and Foxp3^−^ CCR6^+^ CD39^+^ Tregs are the predominant source of IL-10, thus contributing to the amelioration of CIA subsequent LL-IL35 treatment. Such finding may mimic what is evident with human peripheral blood CCR6^+^ CD39^+^ Tregs (64) and CCR6^+^ Tregs found in patients with glomerulonephritis (65).

The data presented demonstrate the potency of IL-35 as an anti-inflammatory therapeutic. Moreover, this investigation further supports the multifaceted benefits of adapting recombinant *L. lactis* as a vector to deliver therapeutic doses of IL-35. In fact, previous studies by us (48) or others, using IL-35 to treat type 1 diabetes model (51), IBD (52), or psoriasis (52), required daily treatments with recombinant protein to control disease. In contrast, only two or three oral doses of LL-IL35 were sufficient to prevent the onset or stop CIA progression. Oral dosing has the substantial advantage of being less invasive circumventing the need for injections. LL-derived IL-35 eliminates the labor-intensive efforts needed to produce and purify the recombinant protein, dramatically reducing the cost of manufacturing this therapeutic. Moreover, IL-35 is a dimeric protein which adds to the difficulty and cost to generate. The *L. lactis* used for this study is a lab-adapted recombinant strain, originally derived from an industrial dairy strain capable of fermenting milk into a product that has the same textural and olfactory properties of yogurt (40). We previously have demonstrated that the curative properties of our recombinant *L. lactis* are maintained when grown on a synthetic medium or used to ferment milk into a yogurt-like product (40). These attributes make *L. lactis* an ideal tolerogen delivery platform for the treatment of autoimmune diseases.

## Author contributions

MM, IK, and DP: conceptualization. MM, IK, CH, and DP: formal analysis, investigation, methodology, validation, visualization, and writing. DP: funding acquisition. All authors approved the final version to be published and agreed to the content and all aspects of the work ensuring the accuracy or integrity of the work are appropriately investigated and presented.

### Conflict of interest statement

The authors declare that the research was conducted in the absence of any commercial or financial relationships that could be construed as a potential conflict of interest.

## References

[1] Hunter TM Boytsov NN Zhang X Schroeder K Michaud K Araujo AB Prevalence of rheumatoid arthritis in the United States adult population in healthcare claims databases, 2004-2014. Rheumatol Int. (2017) 37:1551–7. 10.1007/s00296-017-3726-1 28455559

[2] Cross M Smith E Hoy D Carmona L Wolfe F Vos T The global burden of rheumatoid arthritis: estimates from the Global Burden of Disease 2010 study. Ann Rheum Dis. (2014) 73:1316–22. 10.1136/annrheumdis-2013-204627 24550173

[3] Barbour KE Helmick CG Boring M Brady TJ Prevalence of doctor diagnosed arthritis and arthritis attributable activity limitation—United States, 2013–2015. MMWR Morb Mortal Wkly Rep. (2017) 66:246–53. 10.15585/mmwr.mm6609e1 28278145 PMC5687192

[4] Carmona L Cross M Williams B Lassere M March L Rheumatoid arthritis. Best Pract Res Clin Rheumatol. (2010) 24:733–45. 10.1016/j.berh.2010.10.001 21665122

[5] Scott DL Wolfe F Huizinga TW Rheumatoid arthritis. Lancet (2012) 376:1094–8. 10.1016/S0140-6736(10)60826-4 20870100

[6] de Lange-Brokaar BJ Ioan-Facsinay A van Osch GJ Zuurmond AM Schoones J Synovial inflammation, immune cells and their cytokines in osteoarthritis: a review. Osteoarthritis Cartilage (2012) 12:1484–99. 10.1016/j.joca.2012.08.027 22960092

[7] Jung SM Kim KW Yang CW Park SH Ju JH Cytokine-mediated bone destruction in rheumatoid arthritis. J Immunol Res. (2014) 2014:263625 10.1155/2014/263625 25295284 PMC4176903

[8] Hegen M Keith JC Jr Collins M Nickerson-Nutter CL Utility of animal models for identification of potential therapeutics for rheumatoid arthritis. Ann Rheum Dis. (2008) 67:1505–15. 10.1136/ard.2007.076430 18055474

[9] Saxne T Palladino MA Jr Heinegard D Talal N Wollheim F.A. Detection of tumor necrosis factor alpha but not tumor necrosis factor beta in rheumatoid arthritis synovial fluid and serum. Arithitis Rheum. (1988) 31:1041–5. 10.1002/art.1780310816 3136775

[10] Firestein GS Alvaro-Gracia JM Maki R Alvaro-Garcia JM Quantitative analysis of cytokine gene expression in rheumatoid arthritis. J. Immunol. (1990) 144:3347–53. 2109776

[11] Brand DD Kang AH Rosloniec EF The mouse model of collagen-induced arthritis. Methods Mol. Med. (2004) 102:295–312. 10.1385/1-59259-805-6:295 15286392

[12] Kannan K Ortmann RA Kimpel D Animal models of rheumatoid arthritis and their relevance to human disease. Pathophysiology (2005) 12:167–81. 10.1016/j.pathophys.2005.07.011 16171986

[13] Hu Y Cheng W Cai W Yue Y Li J Zhang P Advances in research on animal models of rheumatoid arthritis. Clin Rheumatol. (2013) 32:161–5. 10.1007/s10067-012-2041-1 22885986

[14] Kourbeti IS Ziakas PD Mylonakis E Biologic therapies in rheumatoid arthritis and the risk of opportunistic infections: a meta-analysis. Clin Infect Dis. (2014) 58:1649–57. 10.1093/cid/ciu185 24647016

[15] Rosenblum H Amita H Anti-TNF therapy: safety aspects of taking the risk. Autoimmun Rev. (2011) 10:563–8. 10.1016/j.autrev.2011.04.010 21570495

[16] Keyser FD Choice of biologic therapy for patients with rheumatoid arthritis: the infection perspective. Curr Rheumatol Rev. (2011) 7:77–87. 10.2174/157339711794474620 22081766 PMC3182090

[17] Yamashita M Ukibe K Matsubara Y Hosoya T Sakai F Kon S *Lactobacillus helveticus* SBT2171 attenuates experimental autoimmune encephalomyelitis in mice. Front Microbiol. (2018) 8:2596 10.3389/fmicb.2017.02596 29403442 PMC5786557

[18] Schorpion A Kolasinski SL Can probiotic supplements improve outcomes in rheumatoid arthritis? Curr Rheumatol Rep. (2017) 19:73 10.1007/s11926-017-0696-y 29094223

[19] Richards JL Yap YA McLeod KH Mackay CR Mariño E Dietary metabolites and the gut microbiota: an alternative approach to control inflammatory and autoimmune diseases. Clin Transl Immunol. (2016) 5:e82 10.1038/cti.2016.29 PMC4910123 27350881

[20] Kandasamy S Vlasova AN Fischer DD Chattha KS Shao L Kumar A Unraveling the differences between gram-positive and gram-negative probiotics in modulating protective immunity to enteric infections. Front Immunol. (2017) 8:334 10.3389/fimmu.2017.00334 28396664 PMC5366325

[21] Sonnenborn U *Escherichia coli* strain Nissle 1917-from bench to bedside and back: history of a special *Escherichia coli* strain with probiotic properties. FEMS Microbiol Lett. (2016) 363:fnw212 10.1093/femsle/fnw212 27619890

[22] Ou B Yang Y Tham WL Chen L Guo J Zhu G Genetic engineering of probiotic *Escherichia coli* Nissle 1917 for clinical application. Appl Microbiol Biotechnol. (2016) 100:8693–9. 10.1007/s00253-016-7829-5 27640192

[23] Galen JE Curtiss R 3rd The delicate balance in genetically engineering live vaccines. Vaccine (2014) 32:4376–85. 10.1016/j.vaccine.2013.12.026 24370705 PMC4069233

[24] Clark-Curtiss JE Curtiss R 3rd *Salmonella* vaccines: conduits for protective antigens. J Immunol. (2018) 200:39–48. 10.4049/jimmunol.1600608 29255088

[25] Kirmiz N Robinson RC Shah IM Barile D Mills DA Milk glycans and their interaction with the infant-gut microbiota. Annu Rev Food Sci Technol. (2018) 9:429–50. 10.1146/annurev-food-030216-030207 29580136 PMC5999319

[26] Moré MI Vandenplas Y *Saccharomyces boulardii* CNCM I-745 improves intestinal enzyme function: a trophic effects review. Clin Med Insights Gastroenterol. (2018) 11:1179552217752679 10.1177/1179552217752679 29449779 PMC5808955

[27] Mays ZJ Nair NU Synthetic biology in probiotic lactic acid bacteria: at the frontier of living therapeutics. Curr Opin Biotechnol. (2018) 53:224–31. 10.1016/j.copbio.2018.01.028 29550614 PMC6139064

[28] Kong W Blanchard AE Liao C Lu T Engineering robust and tunable spatial structures with synthetic gene circuits. Nucleic Acids Res. (2017) 45:1005–14. 10.1093/nar/gkw1045 27899571 PMC5314756

[29] Desmond C Fitzgerald GF Stanton C Ross RP Improved stress tolerance of GroESL-overproducing *Lactococcus lactis* and probiotic *Lactobacillus paracasei* NFBC 338. Appl Environ Microbiol. (2004) 70:5929–36. 10.1128/AEM.70.10.5929-5936.2004 15466535 PMC522070

[30] Mansour NM Abdelaziz SA Oral immunization of mice with engineered *Lactobacillus gasseri* NM713 strain expressing *Streptococcus pyogenes* M6 antigen. Microbiol Immunol. (2016) 60:527–32. 10.1111/1348-0421.12397 27301486

[31] O'Flaherty S Klaenhammer TR Multivalent chromosomal expression of the *Clostridium botulinum* serotype A neurotoxin heavy-chain antigen and the *Bacillus anthracis* protective antigen in *Lactobacillus acidophilus*. Appl Environ Microbiol. (2016) 82:6091–101. 10.1128/AEM.01533-16 27496774 PMC5068166

[32] Li Y Li X Liu H Zhuang S Yang J Zhang F Intranasal immunization with recombinant Lactococci carrying human papillomavirus E7 protein and mouse interleukin-12 DNA induces E7-specific antitumor effects in C57BL/6 mice. Oncol Lett. (2014) 7:576–82. 10.3892/ol.2013.1743 24396491 PMC3881950

[33] Carvalho RDO do Carmo FLR de Oliveira Junior A Langella P Chatel JM Bermúdez-Humarán LG Use of wild type or recombinant lactic acid bacteria as an alternative treatment for gastrointestinal inflammatory diseases: a focus on inflammatory bowel diseases and mucositis. Front Microbiol. (2017) 8:800 10.3389/fmicb.2017.00800 28536562 PMC5422521

[34] Del Carmen S de Moreno de LeBlanc A Martin R Chain F Langella P Bermúdez-Humarán LG Genetically engineered immunomodulatory *Streptococcus thermophilus* strains producing antioxidant enzymes exhibit enhanced anti-inflammatory activities. Appl Environ Microbiol. (2014) 80:869–77. 10.1128/AEM.03296-13 24242245 PMC3911219

[35] Zhang B Li A Zuo F Yu R Zeng Z Ma H Recombinant *Lactococcus lactis* NZ9000 secretes a bioactive kisspeptin that inhibits proliferation and migration of human colon carcinoma HT-29 cells. Microb Cell Fact. (2016) 15:102 10.1186/s12934-016-0506-7 27287327 PMC4901401

[36] Durrer KE Allen MS Hunt von Herbing I Genetically engineered probiotic for the treatment of phenylketonuria (PKU); assessment of a novel treatment *in vitro* and in the PAHenu2 mouse model of PKU. PLoS ONE (2017) 12:e0176286 10.1371/journal.pone.0176286 28520731 PMC5435137

[37] Shigemori S Shimosato T Applications of genetically modified immunobiotics with high immunoregulatory capacity for treatment of inflammatory bowel diseases. Front Immunol. (2017) 8:22 10.3389/fimmu.2017.00022 28179904 PMC5263139

[38] Cano-Garrido O Seras-Franzoso J Garcia-Fruitós E Lactic acid bacteria: reviewing the potential of a promising delivery live vector for biomedical purposes. Microb Cell Fact. (2015) 14:137. 26377321 10.1186/s12934-015-0313-6 PMC4573465

[39] Microbiome Therapeutics and Diagnostics Market 2017-2030. Available online at: https://www.businesswire.com/news/home/20170829005566/en/Microbiome-Therapeutics-Diagnostics-Market-2017-2030—Research

[40] Maddaloni M Kochetkova I Jun S Callis G Thornburg T Pascual DW Milk-based nutraceutical for treating autoimmune arthritis via the stimulation of IL-10- and TGF-ß-producing CD39^+^ regulatory T cells. PLoS ONE (2015) 10:e0117825 10.1371/journal.pone.0117825 25629976 PMC4309564

[41] McLean MH Andrews C Hanson ML Baseler WA Anver MR Senkevitch E Interleukin-27 is a potential rescue therapy for acute severe colitis through interleukin-10-dependent, T-cell-independent attenuation of colonic mucosal innate immune responses. Inflamm Bowel Dis. (2017) 23:1983–95. 10.1097/MIB.0000000000001274 29019857 PMC5796760

[42] Braat H Rottiers P Hommes DW Huyghebaert N Remaut E Remon JP A phase I trial with transgenic bacteria expressing interleukin-10 in Crohn's disease. Clin Gastroenterol Hepatol. (2006) 4:754–9. 10.1016/j.cgh.2006.03.028 16716759

[43] Steidler L Neirynck S Huyghebaert N Snoeck V Vermeire A Goddeeris B Biological containment of genetically modified *Lactococcus lactis* for intestinal delivery of human interleukin 10. Nat Biotechnol. (2003) 21:785–9. 10.1038/nbt840 12808464

[44] Huang A Cheng L He M Nie J Wang J Jiang K Interleukin-35 on B cell and T cell induction and regulation. J Inflamm (Lond). (2017) 14:16 10.1186/s12950-017-0164-5 28794689 PMC5547520

[45] Collison LW Delgoffe GM Guy CS Vignali KM Chaturvedi V Fairweather D The composition and signaling of the IL-35 receptor are unconventional. Nat Immunol. (2012) 13:290–9. 10.1038/ni.2227 22306691 PMC3529151

[46] Wang RX Yu CR Dambuza IM Mahdi RM Dolinska MB Sergeev YV Interleukin-35 induces regulatory B cells that suppress autoimmune disease. Nat Med. (2014) 20:633–41. 10.1038/nm.3554 24743305 PMC4048323

[47] Niedbala W Wei XQ Cai B Hueber AJ Leung BP McInnes IB IL-35 is a novel cytokine with therapeutic effects against collagen-induced arthritis through the expansion of regulatory T cells and suppression of Th17 cells. Eur J Immunol. (2007) 37:3021–9. 10.1002/eji.200737810 17874423

[48] Kochetkova I Golden S Holderness K Callis G Pascual DW IL-35 stimulation of CD39^+^ regulatory T cells confers protection against collagen II-induced arthritis via the production of IL-10. J Immunol. (2010) 184:7144–53. 10.4049/jimmunol.0902739 20483737 PMC3145775

[49] Collison LW Chaturvedi V Henderson AL Giacomin PR Guy C Bankoti J IL-35-mediated induction of a potent regulatory T cell population. Nat Immunol. (2010) 11:1093–101. 10.1038/ni.1952 20953201 PMC3008395

[50] Tirotta E Duncker P Oak J Klaus S Tsukamoto MR Gov L Epstein-Barr virus-induced gene 3 negatively regulates neuroinflammation and T cell activation following coronavirus-induced encephalomyelitis. J Neuroimmunol. (2013) 254:110–6. 10.1016/j.jneuroim.2012.10.005 23102608 PMC3534940

[51] Singh K Kadesjö E Lindroos J Hjort M Lundberg M Espes D Interleukin-35 administration counteracts established murine type 1 diabetes–possible involvement of regulatory T cells. Sci Rep. (2015) 5:12633 10.1038/srep12633 26224624 PMC4519737

[52] Wang Y Mao Y Zhang J Shi G Cheng L Lin Y IL-35 recombinant protein reverses inflammatory bowel disease and psoriasis through regulation of inflammatory cytokines and immune cells. J Cell Mol Med. (2018) 22:1014–25. 10.1111/jcmm.13428 29193791 PMC5783847

[53] Kleinewietfeld M Puentes F Borsellino G Battistini L Rötzschke O Falk K CCR6 expression defines regulatory effector/memory-like cells within the CD25^+^CD4^+^ T-cell subset. Blood (2005) 105:2877–86. 10.1182/blood-2004-07-2505 15613550

[54] Rivino L Gruarin P Häringer B Steinfelder S Lozza L Steckel B CCR6 is expressed on an IL-10-producing, autoreactive memory T cell population with context-dependent regulatory function. J Exp Med. (2010) 207:565–77. 10.1084/jem.20091021 20194631 PMC2839148

[55] Li N Wei W Yin F Chen M Ma TR Wu Q The abnormal expression of CCR4 and CCR6 on Tregs in rheumatoid arthritis. Int J Clin Exp Med. (2015) 8:15043–53. 26628988 PMC4658877

[56] Smelt MJ de Haan BJ Bron PA van Swam I Meijerink M Wells JM *L. plantarum, L. salivarius, and L. lactis* attenuate Th2 responses and increase Treg frequencies in healthy mice in a strain dependent manner. PLoS ONE (2012) 7:e47244 10.1371/journal.pone.0047244 23056616 PMC3467239

[57] Huibregtse IL Snoeck V de Creus A Braat H De Jong EC Van Deventer SJ Induction of ovalbumin-specific tolerance by oral administration of *Lactococcus lactis* secreting ovalbumin. Gastroenterology (2007) 133:517–28. 10.1053/j.gastro.2007.04.073 17681173

[58] Borsellino G Kleinewietfeld M Di Mitri D Sternjak A Diamantini A Giometto R Expression of ectonucleotidase CD39 by Foxp3^+^ Treg cells: hydrolysis of extracellular ATP and immune suppression. Blood (2007) 110:1225–32. 10.1182/blood-2006-12-064527 17449799

[59] Godefroy E Alameddine J Montassier E Mathé J Desfrançois-Noël J Marec N Expression of CCR6 and CXCR6 by gut-derived CD4^+^/CD8α^+^ T-regulatory cells, which are decreased in blood samples from patients with inflammatory bowel diseases. Gastroenterology (2018) 155:1205–17. 10.1053/j.gastro.2018.06.078 29981781

[60] Kochetkova I Crist K Callis G Pascual DW Segregated regulatory CD39^+^ CD4^+^ T cell function: TGF-β-producing Foxp3^−^ and IL-10-producing Foxp3^+^ cells are interdependent for protection against collagen-induced arthritis. J Immunol. (2011) 187:4654–66. 10.4049/jimmunol.1100530 21967895 PMC3237119

[61] Kochetkova I Thornburg T Callis G Holderness K Maddaloni M Pascual DW Oral *Escherichia coli* colonization factor antigen I fimbriae ameliorate arthritis via IL-35, not IL-27. J Immunol. (2014) 192:804–16. 10.4049/jimmunol.1302018 24337375 PMC3903302

[62] Rampersad RR Tarrant TK Vallanat CT Quintero-Matthews T Weeks MF Enhanced Th17-cell responses render CCR2-deficient mice more susceptible for autoimmune arthritis. PLoS ONE (2011) 6:e25833 10.1371/journal.pone.0025833 21991368 PMC3186765

[63] Deaglio S Dwyer KM Gao W Friedman D Usheva A Erat A Adenosine generation catalyzed by CD39 and CD73 expressed on regulatory T cells mediates immune suppression. J Exp Med. (2007) 204:1257–65. 10.1084/jem.20062512 17502665 PMC2118603

[64] Magid-Bernstein JR Rohowsky-Kochan CM Human CD39^+^ Treg cells express Th17-associated surface markers and suppress IL-17 via a Stat3-dependent mechanism. J Interferon Cytokine Res. (2017) 37:153–64. 10.1089/jir.2016.0071 28387597 PMC6435349

[65] Kluger MA Luig M Wegscheid C Goerke B Paust HJ Brix SR Stat3 programs Th17-specific regulatory T cells to control GN. J Am Soc Nephrol. (2014) 25:1291–302. 10.1681/ASN.2013080904 24511136 PMC4033381

